# Health literacy, Trust in doctors, Acculturation - analysis of patient satisfaction of Arab immigrants in general medical care in Germany

**DOI:** 10.1186/s12913-026-14675-z

**Published:** 2026-05-05

**Authors:** Sara Kesserawi, Jutta Hübner, Emadaldin Ahmadi

**Affiliations:** Klinik für Innere Medizin II, Universitätsklinikum Jena, Comprehensive Cancer Center Central Germany – Campus Jena, Am Klinikum 1, 07747 Jena, Germany

**Keywords:** Health literacy, Patient satisfaction, Trust in doctors, Acculturation, Arab immigrants, Migrant health, Primary care, Germany

## Abstract

Patient satisfaction is a crucial measure of healthcare quality, especially for Arab immigrants who often face barriers in accessing care. We investigated in this study patient satisfaction among Arab immigrants in Germany’s general medical care system, focusing on the roles of trust in doctors, health literacy, and acculturation. Using a cross-sectional online survey, we collected data from 165 eligible participants whohad previously consulted a doctors in Germany. We used validated instruments to assess patient satisfaction (EUROPEP), trust in doctors, health literacy, and acculturation. We examined associations between these variables using correlation and multiple linear regression analyses. Overall patient satisfaction was moderate (mean score: 78.5 ± 18.1), with particular dissatisfaction reported regarding accessibility issues like appointment scheduling and waiting times. Trust in doctors emerged as the strongest predictor of patient satisfaction (β = 0.82, *p* < 0.001). Higher levels of health literacy (β = −0.16, *p* < 0.001) and acculturation (β = −0.12, *p* = 0.003) were independently associated with lower satisfaction. The psychosocial regression model explained a substantial proportion of variance in patient satisfaction (R² = 0.76). In contrast, sociodemographic variables, including gender, education, and duration of residence in Germany, were not significantly associated with patient satisfaction.These findings highlight the importance of trust-building and effective communication in healthcare for migrant populations. The findings underscore the relevance of culturally sensitive healthcare approaches that address both interpersonal and systemic factors to improve patient experiences for Arab immigrants in Germany.

**Supplementary Information:**

The online version contains supplementary material available at 10.1186/s12913-026-14675-z.

## Introduction

Patient satisfaction is widely seen as a key measure of healthcare quality and is connected to better patient safety, more effective clinical care, improved treatment adherence, and increased involvement in shared decision-making [1]. Greater satisfaction is linked to better communication between doctors and patients, higher levels of trust, and stronger therapeutic relationships. In Germany, patient satisfaction has become a significant area of focus in health services research, and results from the EUROPEP study have shown generally high levels of satisfaction with primary care among the general public [2]. However, these overall findings might hide differences in healthcare experiences among people with a migration background.

Over the last thirty years, Germany has experienced significant demographic changes due to migration. From 1991 to 2020, net migration amounted to about 8.9 million people [3]. By 2022, roughly 24.9 million residents had a migration background, including approximately 1.3 million individuals from Syria, who form the largest Arab migrant group in the country [4]. Despite this population shift, Arab immigrants are still underrepresented in health services research and are frequently grouped into broader migrant categories. This classification hinders the ability to recognize specific factors that influence their healthcare experiences.

Crucially, the Arab immigrant community in Germany is very diverse. Migration routes encompass humanitarian migration, family reunification, educational migration, and employment-based migration. Especially among Syrian migrants, experiences of being forced to leave their homes and having refugee status can influence their expectations from healthcare systems, their perception of barriers to access, and their level of trust in institutions. These variations show that patient satisfaction should not be viewed only as a personal assessment of care, but needs to be considered within wider social, cultural, and structural contexts.

**Fig. 1:**
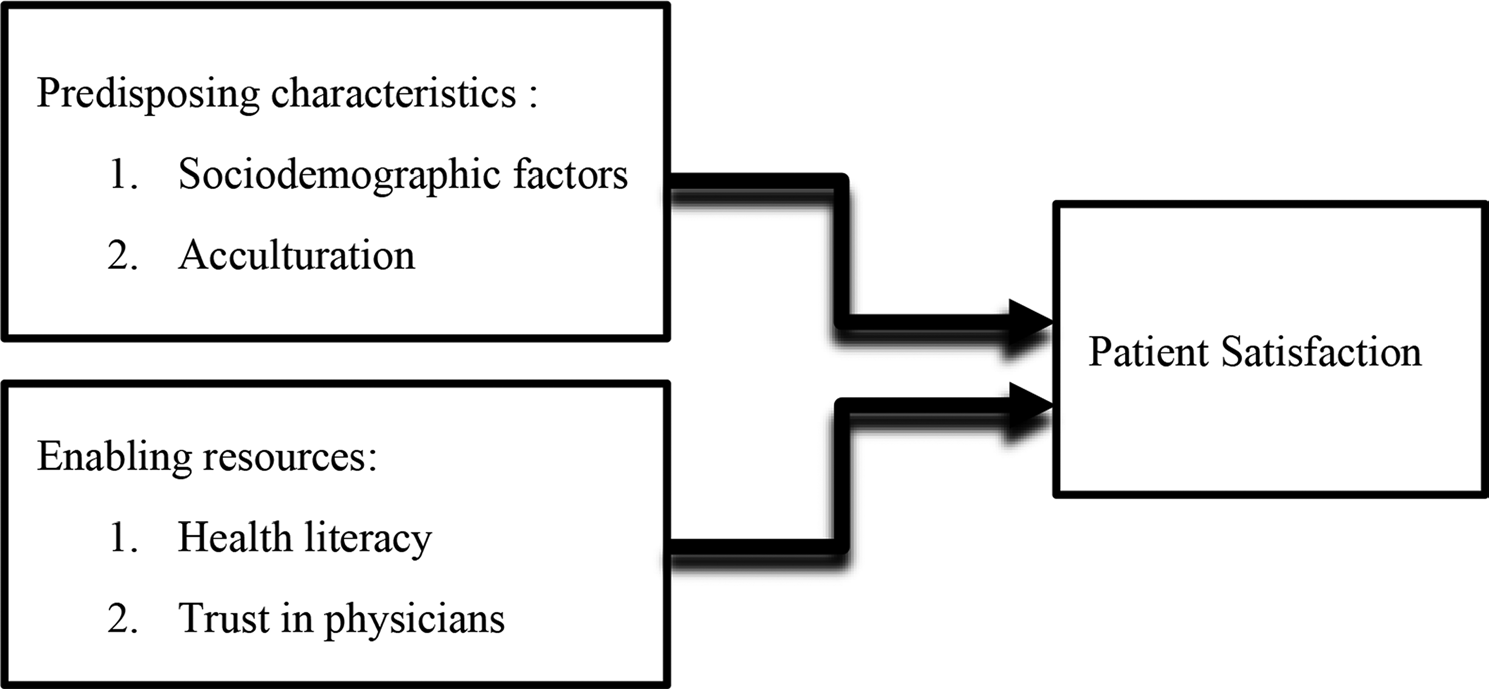
Conceptual framework of the study based on Andersen’s Behavioral Model of Health Services Use

To provide a conceptual framework for these processes, the current study utilizes Andersen’s Behavioral Model of Health Services Use (see Fig. 1). This model explains healthcare use and its outcomes as arising from the interaction of predisposing traits, enabling resources, and need-related factors at both individual and systemic levels. Predisposing traits involve sociodemographic factors and cultural orientation, while enabling resources refer to the capabilities that help individuals navigate and engage with healthcare systems. In the present study, sociodemographic variables and acculturation are conceptualized as predisposing factors, whereas health literacy and interpersonal trust in physicians are treated as enabling resources.Within this model, patient satisfaction is seen as a subjective result influenced not only by personal interactions but also by structural and institutional factors that affect access to and the delivery of care.

Using this framework to examine the healthcare experiences of migrants indicates that satisfaction is shaped by both consistent background traits and changeable psychosocial factors. Sociodemographic factors may represent an individual’s structural position in society, while psychosocial elements like health literacy, cultural adaptation, and confidence in healthcare providers reflect closer processes through which people understand and assess their healthcare experiences.

In addition, Berry’s acculturation framework provides a complementary perspective by distinguishing between different strategies of cultural adaptation (e.g., integration, assimilation, separation, marginalization). Although the present study did not differentiate between these strategies, Berry’s model offers a conceptual lens for understanding how varying patterns of cultural orientation may influence trust and satisfaction within healthcare contexts.

An increasing amount of European research suggests that individuals with a migration background tend to express lower levels of satisfaction with healthcare services compared to those without such a background [5–11]. For instance, studies conducted in Denmark and Finland have identified particularly low satisfaction rates among Middle Eastern and African migrant communities [12, 13]. Language difficulties play a significant role in this dissatisfaction, as they can lead to communication problems, incorrect diagnoses, and poor compliance with medical advice [8, 14, 15]. Additionally, cultural misunderstandings, unfulfilled religious requirements, and lack of familiarity with healthcare practices further influence how patients perceive the quality of their care [5, 16, 17].

Health literacy serves as a crucial enabling factor in Andersen’s model. It is defined as the capacity to obtain, comprehend, evaluate, and use health-related information, which affects how patients interact with healthcare systems and make medical decisions. The Federal Center for Health Education (BZgA) views health literacy as a complex concept influenced by cognitive, psychological, and environmental elements [18]. In Germany, health literacy varies significantly, with lower levels observed among individuals from socioeconomically disadvantaged backgrounds and those with less education [19, 20]. Limited health literacy is linked to decreased involvement in preventive care, increased use of healthcare services, and poorer control of chronic illnesses [21–23]. Earlier studies have typically found a positive relationship between health literacy and patient satisfaction [24], indicating that more informed patients may feel more empowered and assured during healthcare encounters.

Acculturation—the process of adapting to the cultural norms of a host society—constitutes another relevant predisposing factor. Higher levels of acculturation have been associated with improved access to preventive services, increased healthcare utilization, and stronger integration into healthcare systems [25]. Acculturation has also been linked to greater trust in physicians [26] and, in some studies, to higher levels of medical satisfaction [27]. Trust in doctors, particularly interpersonal trust in individual providers, is a well-established determinant of patient satisfaction [28, 29]. Trust facilitates open communication, enhances adherence to medical advice, and reduces perceived uncertainty. At the same time, migrant families have been shown to report lower levels of trust in doctors compared with majority populations [30], potentially contributing to lower satisfaction. In this study, trust refers specifically to interpersonal trust in individual doctors, rather than institutional trust in the healthcare system, which represents a distinct but related construct.

Taken together, the existing literature suggests that patient satisfaction among migrants arises from a multifaceted interplay between informational resources, cultural adaptation processes, and interpersonal trust in healthcare providers. However, most prior studies have examined these determinants in isolation, without integrating them within a coherent theoretical framework. In particular, there is a lack of empirical research simultaneously investigating health literacy, acculturation, and trust within Andersen’s Behavioral Model of Health Services Use among Arab immigrants in Germany. This gap limits a comprehensive understanding of how structural position and psychosocial resources jointly shape satisfaction outcomes in this rapidly growing population.

Against this background, the present study systematically examines patient satisfaction among Arab immigrants in Germany’s primary care system within a theory-driven framework. Guided by Andersen’s model, we analyze the relative contributions of sociodemographic characteristics and psychosocial factors to patient satisfaction using multivariate regression analyses. Specifically, we assess whether health literacy, acculturation, and interpersonal trust in physicians independently predict satisfaction levels and whether these psychosocial determinants explain a greater proportion of variance than sociodemographic variables. By integrating individual-level resources with structural considerations, this study aims to advance the theoretical understanding of migrant healthcare experiences and to inform culturally responsive primary care strategies.

## Methods

### Study design and participants

We conducted a cross-sectional study using an online survey to evaluate patient satisfaction, trust in doctors, health literacy, and acculturation among Arab immigrants in Germany. We recruited participants using a snowball sampling approach with multiple entry points. Initial invitations were distributed through migrant community organizations, municipal integration offices, refugee support initiatives, and social media channels targeting Arab communities in Germany. Recruitment did not rely on a single referral chain, and the number of initial seed participants was therefore not formally recorded. This approach may have favored participation by individuals who were more educated, digitally connected, or engaged with community organizations.

We hosted the survey on the SoSci survey platform and made available in both German and Arabic to accommodate participants’ language preferences.

We translated the questionnaire from German into Arabic by the first author, a native Arabic speaker with familiarity with health-related terminology and the study content. Following translation, the Arabic version was reviewed through informal pretesting with Arabic-speaking individuals to assess clarity, comprehensibility, and linguistic appropriateness of the items. Feedback from this process was used to refine wording where necessary.

Inclusion criteria:

1. Self-identified Arab immigrants (holding Arab nationality) residing in Germany.
2. Age ≥ 18 years.
3. holding a valid residence permit.
4. having had at least one medical consultation in Germany.

Data collection took place from May to August 2024. Of the 319 individuals who accessed the survey, 175 completed the questionnaire. Ten respondents did not meet the predefined inclusion criteria and were excluded, resulting in a final analytical sample of 165 participants.

Questionnaires and Measures:

A standardized questionnaire was developed using four validated instruments:

1. EUROPEP: Assessed patient satisfaction with primary care [2].
2. Glattacker et al. questionnaire: Measured trust in doctors [31].
3. Mähler et al. questionnaire: Evaluated acculturation [32].
4. HLS-EU-Q16: Quantified health literacy [33, 34].

### Survey administration and data collection

The survey was administered using SoSci Survey, a secure web-based platform for academic research.

Participants accessed the survey through unique links distributed via professional networks (including migrant community organizations and doctors) and community channels (such as social media groups and cultural associations). Prior to participation, all respondents provided digital informed consent after reviewing detailed information about the study’s purpose, data anonymity guarantees, and privacy protection measures. The survey collected sociodemographic information, and four validated scales were used to assess patient satisfaction (EUROPEP), Trust in doctors (Glattacker scale), health literacy (HLS-EU-Q16), acculturation [2, 31, 32, 34]. 

Following data collection, all responses were automatically anonymized and securely exported in encrypted format for analysis. As the survey link was openly distributed, complete prevention of duplicate entries across different devices or browsers cannot be guaranteed. Therefore, the dataset was checked for potential duplicate responses by examining response patterns and completion times during data cleaning. In addition, responses were inspected for obvious indicators of low data quality (e.g., straight-lining or implausibly short completion times). No problematic cases were identified and retained for analysis.

### Instruments

The questionnaire included four validated instruments assessing patient satisfaction, trust in doctors, health literacy, and acculturation.

*Patient satisfaction* was evaluated using the 23-item EUROPEP questionnaire, which examines five critical domains of primary care: (1) doctor-patient relationship (including time allocation, active listening, and shared decision-making), (2) medical/technical care (covering preventive services and clinical competence), (3) support staff professionalism, (4) practice organization (accessibility and wait times), and (5) overall satisfaction. Participants rated their experiences over the preceding 12 months on a 5-point Likert scale ranging from “poor” [1] to “excellent” [35, 36].

*Trust in doctors* was measured through the German version of the Trust in doctors Scale, an 11-item instrument assessing three key dimensions: reliability of care, fiduciary confidence, and maintenance of confidentiality. Responses were collected using a 5-point Likert scale from “strongly disagree” to “strongly agree” [31].

*Health literacy* assessment utilized the HLS-EU-Q16 [33, 34], a 16-item short form evaluating four core competencies: (1) accessing, (2) understanding, (3) appraising, and (4) applying health information across prevention, promotion, and healthcare contexts.

Participants rated their perceived difficulty for each item on a 4-point scale from “very easy” to “very difficult” [33, 34].

*Acculturation* was examined through a 20-item scale measuring behavioral adaptation, sociocultural adjustment, and social integration interest. All items employed a 5- point response scale ranging from “does not apply at all” [1] to “applies completely” [32, 35].

We assessed scale reliability using Cronbach’s α (α = 0.815–0.963), indicating good to excellent internal consistency and supporting the adequate performance of the translated instruments, Cronbach’s α values for each instrument are presented in Table 1.

**Table 1:** Internal consistency of study instruments

Scale	Number of Items	Cronbach’s alpha
Patient Satisfaction (EUROPEP)	23	0.963
Trust in doctors	11	0.904
Health Literacy (HLS_EU_Q16)	16	0.898
Acculturation	20	0.815

### Statistical analysis

We used descriptive statistics (frequencies, percentages, means, and histograms) to describe the sample’s sociodemographic characteristics and the distribution of scale scores.

We conducted Pearson correlation analyses to examine associations between acculturation, health literacy, trust in doctors, and patient satisfaction.

To evaluate predictors of patient satisfaction, two separate multiple linear regression models were specified for sociodemographic and psychosocial variables. Sociodemographic variables represent relatively stable background characteristics, whereas psychosocial constructs such as trust in doctors, health literacy, and acculturation capture proximal mechanisms more directly related to patients’ healthcare experiences. Combining these variables within a single model could result in overadjustment, because duration of residence and other sociodemographic characteristics are conceptually and empirically related to acculturation and health literacy and may lie on the same causal pathway. Accordingly, specifying separate models allows a clearer interpretation of the unique contribution of psychosocial predictors to patient satisfaction while reducing the risk of statistical overcontrol.

For regression and correlation analyses, cases with missing values on any of the variables included in the respective models were excluded using listwise deletion. No imputation procedures were applied.

The adequacy of the sample size for multiple regression was evaluated based on Green’s (1991) recommendations. For testing individual predictors (*N* ≥ 104 + m), where m represents the number of predictors in the model, a minimum of 107 cases was required for the psychosocial model including three predictors. With a final sample of 165 participants, this criterion was exceeded [37].

We evaluated regression assumptions using graphical diagnostics. Normality of residuals was assessed using histograms and normal Q–Q plots. Homoscedasticity was examined by visual inspection of scatterplots of standardized residuals versus standardized predicted values. Multicollinearity was assessed using variance inflation factors (VIF).All assumptions were met (VIF < 2.0).

Duration of residence in Germany was coded as an ordered variable (< 5, 5–10, 10–25, > 25 years) and entered accordingly. Sensitivity analyses additionally adjusted for residence status (humanitarian vs. non-humanitarian), country of birth (Syria vs. other Arab countries), and duration of residence to assess the robustness of the findings.

Duration of residence was additionally included as a covariate in a sensitivity analysis of the psychosocial model.

All analyses were conducted using IBM SPSS Statistics Version 25.

#### Ethics

This study was reviewed and approved by the Ethics Committee of the Medical Faculty at Friedrich Schiller University. Participation in the survey was voluntary, and all responses were collected anonymously. Before participating, respondents were informed about the purpose of the study, their right to withdraw at any time, and how their data would be handled in accordance with data protection regulations.

## Results

Of the 319 individuals who accessed the online survey, 165 met all inclusion criteria and were included in the analysis. Overall, the sample was relatively young and predominantly female, with most participants having lived in Germany for several years. Most respondents were first-generation immigrants, and the majority were born in Syria. Detailed sociodemographic characteristics are summarized in Table 2, and countries of birth are presented in Table 3.

**Table 2:** Demographic and survey characteristics (*N* = 165)

Characteristic	Frequency (%)
Age (years)	
18–40	130 (78.8%)
41–59	29 (17.6%)
≥ 60	6 (3.6%)
Gender	
Female	102 (61.8%)
Male	60 (36.4%)
Gender Diverse	3 (1.8%)
Years in Germany	
< 5	19 (11.5%)
5–10	104 (63.0%)
10–15	26 (15.8%)
> 25	16 (9.7%)
Immigrated to Germany	
Yes	154 (93.3%)
No	11 (6.7%)
Residence Status	
Humanitarian	37 (22.4%)
Family	14 (8.5%)
Education	12 (7.3%)
Employment	6 (3.6%)
EU Blue Card	1 (0.6%)
Permanent	14 (8.5%)
Citizenship	78 (47.3%)
Education	
University of applied sciences	108 (65.5%)
Graduation after 8/10 years	8 (4.8%)
Abitur	43 (26.1%)
No degree	6 (3.6%)
Religion	
Muslim	148 (65.5%)
Christian	5 (3.0%)
Other	2 (1.2%)
None	10 (6.1%)
Marital Status	
Married	100 (60.6%)
Single	53 (32.1%)
Divorced	4 (2.4%)
In a relationship	7 (4.2%)
Widowed	1 (0.6%)
Financial Satisfaction	
Quite satisfied	108 (65.5%)
Very dissatisfied	18 (10.9%)
Dissatisfied	6 (3.6%)
Quite dissatisfied	33 (20.0%)

**Table 3:** Country of birth of participants (*N* = 165)

Country of Birth	Frequency (*n*)	Percent (%)
Syria (all variations)	122	73.9
Egypt	6	3.6
Lebanon	5	3.0
Palestine	3	1.8
Iraq	2	1.2
Jordan	2	1.2
Saudi Arabia	3	1.8
UAE	2	1.2
Libya	1	0.6
Morocco	1	0.6
Tunisia	1	0.6
Qatar	1	0.6
Cyprus	1	0.6
Poland	1	0.6
No response	14	8.5
Total	165	100.0

### Correlation analysis

We calculated Pearson correlation coefficients to examine the strength and direction of associations among Trust in Doctors, Patient Satisfaction, Health Literacy, and Acculturation (Table 4). A nearly perfect positive relationship emerged between Trust in Doctors and Patient Satisfaction (*r* = 0.855, *p* < 0.001), underscoring that as patients’ confidence in their doctors increases, their overall satisfaction with care rises in tandem.

Health Literacy demonstrated moderate but statistically significant negative correlations with both Patient Satisfaction (*r* = − 0.453, *p* < 0.001) and Trust in Doctors (*r* = − 0.379, *p* < 0.001). These findings suggest that participants with higher self-reported ability to access, understand, and apply health information may also hold more critical expectations of care, translating into comparatively lower satisfaction and less unconditional trust in their providers.

Acculturation was positively and significantly associated with Trust in Doctors (*r* = 0.228, *p* = 0.003), indicating that greater sociocultural adaptation to the host country corresponds to somewhat higher levels of patient–doctors trust. However, acculturation bore no significant relationship with Health Literacy (*r* = − 0.132, *p* = 0.090) or Patient Satisfaction (*r* = 0.091, *p* = 0.246), implying that while integration may bolster interpersonal trust, it does not necessarily influence patients’ informational competencies or satisfaction judgments in isolation.

Overall, these correlation patterns highlight the complex interplay between cognitive competencies (health literacy), cultural integration (acculturation), interpersonal trust, and evaluative judgments of care quality. Based on these observed relationships, two multiple linear regression models were subsequently calculated: one focusing on psychosocial predictors (trust, literacy, acculturation) and the other on sociodemographic characteristics. These models aimed to clarify the strength and direction of each variable’s influence on patient satisfaction.

Descriptive statistics indicated moderate variability across all scales. Skewness and kurtosis values were within acceptable ranges (|skewness| < 1; |kurtosis| < 2), indicating no substantial deviations from normality and supporting the use of parametric regression analyses.

**Table 4:** Descriptive statistics and Pearson correlations between key variables (*N* = 165)

Variable	Mean	SD	Min	Max	Skewness	Kurtosis	1	2	3
Acculturation	69.5	8.9	33	96.	-0.035	1.210	—		
Health Literacy	34.0	7.4	16	59	-0.075	0.412	-0.132 (*p* = 0.090)	—	
Patient satisfaction	78.5	18.1	32	115	-0.296	-0.421	0.091 (*p* = 0.246)	-0.453 (*p* < 0.001)	—
Trust in doctors	35.6	8.4	17	55	-0.020	-0.323	0.228 (*p* = 0.003)	-0.379 (*p* < 0.001)	0.855 (*p* < 0.001)

Note. Values are Pearson correlation coefficients (R) with 2-tailed P-values in parentheses

### Regression analyses

To identify key drivers of patient satisfaction among Arab immigrants in Germany, two separate multiple linear regression models were estimated.

Regression diagnostics indicated no substantial violations of assumptions. Residual plots suggested approximate normality and homoscedasticity, and multicollinearity was low (VIF 1.06–1.21; Durbin–Watson 1.84). Detailed regression assumption diagnostics are presented in Table 5. Detailed diagnostic plots are provided in the Supplementary Materials.

**Table 5:** Regression assumption diagnostics for psychosocial model (*N* = 165)

Parameter	Predictor	Observed value
VIF	Health Literacy	1.18
	Trust in Doctors	1.21
	Acculturation	1.06
	Health Literacy	0.85
	Trust in Doctors	0.82
	Acculturation	0.95
Durbin–Watson	—	1.84

Note. Acceptable thresholds: VIF < 5 (preferably < 2); tolerance > 0.20; Durbin–Watson between 1.5 and 2.5

### Model 1: Psychosocial predictors

This model included Health Literacy, Trust in Doctors, and Acculturation as predictors of patient satisfaction. It accounted for 76.3% of the variance in satisfaction (*R* = 0.873; R² = 0.763; Adjusted R² = 0.758; F(3, 161) = 172.59, *p* < 0.001). Trust in Doctors emerged as a very strong positive predictor (β = 0.822, *p* < 0.001). In contrast, both Health Literacy (β = − 0.157, *p* < 0.001) and Acculturation (β = − 0.117, *p* = 0.003) were negatively associated with satisfaction (Table 6).

In an additional sensitivity analysis, duration of residence in Germany was included as a covariate in the psychosocial regression model. The inclusion of this variable did not materially alter the regression coefficients or significance levels of the psychosocial predictors. Trust in doctors remained strongly positively associated with patient satisfaction, whereas health literacy and acculturation remained negatively associated. Duration of residence itself was not significantly associated with patient satisfaction.

In further sensitivity analyses adjusting for country of birth (Syria vs. other Arab countries), humanitarian residence status was not significantly associated with patient satisfaction. Country of birth itself was also not significantly associated with patient satisfaction, whereas age remained the only sociodemographic variable independently associated with patient satisfaction. The inclusion of country of birth did not materially change the main findings.

The strong association between trust and satisfaction may partly reflect conceptual overlap between relational evaluation and global satisfaction judgments.

**Table 6:** Multiple linear regression predicting Patient satisfaction (psychosocial predictors)

Predictor	Unstandardized regression coefficient (B)	standard errors (SE)	standardized regression coefficients (β)	t-value	*p*-value
(Constant)	44.81	7.52		5.959	< 0.001
Health Literacy	-0.38	0.10	-0.157	-3.780	< 0.001
Trust in Doctors	1.78	0.09	0.822	19.443	< 0.001
Acculturation	-0.24	0.08	-0.117	-2.976	0.003

Note: *R* = 0.873; R² = 0.763; Adjusted R² = 0.758; Std. Error = 8.89

### Model 2: Sociodemographic predictors

Age, gender, years living in Germany, and Education Level explained only 3.5% of the variance (*R* = 0.187; R² = 0.035; Adjusted R² = 0.011; F(4, 160) = 1.45, *p* = 0.222). The only significant sociodemographic predictor was Age (β = 0.180, *p* = 0.024), indicating that older participants reported slightly higher satisfaction. Gender, residence duration, and education level had no meaningful effects (Table 7).

In sensitivity analyses additionally adjusting for country of birth (Syria vs. other Arab countries), humanitarian residence status was not significantly associated with patient satisfaction. Country of birth itself was also not significantly associated with patient satisfaction, whereas age emerged as the only variable independently associated with patient satisfaction. The inclusion of country of birth did not materially change the main findings.

These findings suggest that, despite the heterogeneous migration trajectories within the sample, the observed associations between trust in doctors, health literacy, acculturation, and patient satisfaction were robust across subgroups defined by country of birth and residence status. Thus, the main psychosocial relationships identified in this study do not appear to be driven solely by the overrepresentation of participants of Syrian origin or by humanitarian migration pathways.

Overall, psychosocial factors—especially trust in doctors—play a substantially larger role in shaping patient satisfaction than basic demographic characteristics.

Duration of residence in Germany was not included in the primary psychosocial model to avoid overadjustment, given its close conceptual relationship with acculturation and health literacy, but was examined in a sensitivity analysis to assess potential residual confounding.

**Table 7:** Multiple linear regression predicting Patient satisfaction (sociodemographic predictors)

Predictor	B	SE	β	t	*p*-value
(Constant)	72.13	8.948		8.061	< 0.001
Age	6.366	2.792	0.180	2.280	0.024
Gender	1.241	2.673	0.036	0.464	0.643
Years living in Germany	-0.882	1.831	-0.038	-0.482	0.631
Education level	-0.381	1.879	-0.016	-0.203	0.840

Note: *R* = 0.187; R² = 0.035; Adjusted R² = 0.011; Std. Error = 17.99

Note. B = unstandardized regression coefficient; SE = standard error; β = standardized regression coefficient; t = t-value; p = p-value

## Discussion

This study provides a nuanced understanding of patient satisfaction among Arab immigrants in Germany by demonstrating that, within this sample, satisfaction levels were lower than those reported in the general German population. The mean EUROPEP score in the present study was 78.5 ± 18.1. A direct statistical comparison with the original EUROPEP study is not feasible because the published report primarily provides proportions of positive ratings rather than comparable mean scores and variance estimates. This finding is consistent with extensive European evidence on migrant–non-migrant disparities in healthcare experiences [7, 16]. These differences have been attributed in the literature to factors such as linguistic barriers, cultural misunderstandings, and structural inequities within healthcare systems [5, 8, 14, 16]. In the German healthcare context, health insurance status (private vs. statutory) represents an additional key structural determinant that may influence access to care, waiting times, and perceived quality of services. However, this variable was not assessed in the present study, which limits the extent to which observed differences in patient satisfaction can be fully attributed to psychosocial factors alone, as structural inequalities related to insurance coverage may also contribute to patients’ healthcare experiences.

Among the psychosocial predictors, trust in doctors emerged as the strongest determinant of patient satisfaction (β = 0.822), with an effect size substantially larger than those of health literacy (β = −0.157) and acculturation (β = −0.117). This finding aligns with prior research demonstrating the central role of interpersonal trust in shaping healthcare experiences and satisfaction, particularly among immigrant populations [28, 29, 31]. Trust may function as a potential mechanism in this context that helps patients navigate unfamiliar healthcare systems and interpret care encounters more positively, even in the presence of structural constraints.

Importantly, these patterns should be interpreted in light of the substantial heterogeneity within Arab immigrant populations. The category “Arab immigrants” encompasses diverse migration trajectories, including forced and voluntary migration, as well as differences in legal status, duration of residence, and prior exposure to healthcare systems. These dimensions are likely to shape expectations, trust in healthcare providers, and evaluations of care in ways that cannot be fully captured by broad categorizations.

In the present study, sensitivity analyses were conducted to explore whether patient satisfaction differed according to country of birth or humanitarian residence status. While neither variable was significantly associated with patient satisfaction and did not materially alter the regression coefficients, these analyses do not fully address the underlying conceptual heterogeneity. More fine-grained differences related to migration experiences and social context may remain undetected, particularly in samples of limited size or imbalance.

Consequently, the observed associations should not be interpreted as reflecting a uniform pattern across all individuals classified within this group. Rather, they may obscure important within-group differences that warrant further investigation. Future studies should therefore adopt more differentiated approaches to capturing migration histories and contextual factors.

One of the most theoretically salient findings is the negative association between both health literacy (β = −0.157, *p* < 0.001) and acculturation (β = −0.117, *p* = 0.003) and patient satisfaction. While this finding contrasts with much of the literature that conceptualizes these constructs as facilitators of improved healthcare experiences (e.g., 26,27), it may be better understood within broader theoretical frameworks linking increasing patient agency, expectations, and system evaluation.

From this perspective, several not mutually exclusive mechanisms may help explain the observed associations.

First, the “critical consumer” hypothesis suggests that increasing health literacy and acculturation enhance individuals’ ability and willingness to critically evaluate healthcare systems, potentially resulting in more stringent satisfaction judgments [27]. This interpretation aligns with Han and Lee [27], who found that acculturation can increase patients’ willingness to critically evaluate healthcare systems.

Second, the “expectation-reality gap” theory posits that acculturation exposes immigrants to German healthcare standards without necessarily providing equal access to that standard of care. Such a disparity between expectations and experiences may help to explain lower satisfaction ratings among more acculturated individuals. This phenomenon has been observed in other contexts and may also be relevant here. In contexts where marginalized groups may gain awareness of mainstream standards without concomitant improvements in service provision.

Third, the “systemic frustration” model suggests that prolonged exposure to the healthcare system may reveal persistent structural barriers (e.g., discrimination, bureaucratic obstacles) that may not be immediately visible to less acculturated individuals. This interpretation is consistent with findings by Morawa et al. [38], who reported that certain acculturation patterns are associated with increased psychological distress among Turkish immigrants in Germany.

Taken together, these perspectives converge on the interpretation that increasing health literacy and acculturation may not simply function as facilitators of positive healthcare experiences, but may also heighten patients’ awareness of discrepancies between expectations and system performance. This pattern may be understood within an “informed but structurally constrained patient” perspective, in which increased knowledge and cultural adaptation are accompanied by greater sensitivity to structural limitations in care delivery.

Given the cross-sectional design of the study, alternative causal directions cannot be ruled out. For example, lower patient satisfaction or negative healthcare experiences may also influence how individuals perceive and report their health literacy or acculturation-related experiences.

Notably, these associations persisted after adjustment for duration of residence, suggesting that acculturation and health literacy capture psychosocial processes beyond time spent in the host country.

A further limitation relates to potential residual confounding arising from the close interrelationships between duration of residence, acculturation, and health literacy. Although these constructs were included in separate regression models to reduce multicollinearity and to allow for conceptual differentiation, they are inherently interrelated and may partially capture overlapping underlying processes related to adaptation and familiarity with the healthcare system. As a result, some degree of residual confounding cannot be ruled out. This limitation should be considered when interpreting the independent effects of acculturation and health literacy, as their estimated associations may partially reflect shared variance with time-related adaptation processes.

An additional finding that warrants further consideration is the positive association between age and patient satisfaction observed in the sociodemographic regression model. Older participants reported higher satisfaction levels than younger respondents. Similar age-related patterns have been observed in previous studies of patient satisfaction and healthcare experiences among migrant populations in Germany and other European countries [5, 9]. Several tentative explanations may be considered. Older individuals in this sample may hold lower expectations toward healthcare services or evaluate care more favorably based on comparisons with healthcare systems in their countries of origin, where access and coverage may have been more limited. Moreover, prior research suggests that lower levels of health literacy, which are more prevalent in older age groups, may be associated with less critical evaluations of care. Importantly, the effect of age was no longer prominent once psychosocial factors were considered, suggesting that age-related differences may operate indirectly through expectations, experiences, and trust rather than representing an independent driver of satisfaction.

The high proportion of married participants in the sample may also be relevant for interpreting the findings. Marital status is often associated with greater social support, which may facilitate healthcare navigation and influence perceptions of care quality. As marital status was not included as a predictor in the regression models, its potential role should be interpreted cautiously and may represent an unmeasured contextual factor rather than a direct explanatory variable.

Finally, the relatively high educational level of the sample may have influenced the observed associations. Higher education is closely linked to greater health literacy and may foster more critical expectations toward healthcare services. In this context, the overrepresentation of university-educated participants may partially contribute to the negative association between health literacy and patient satisfaction observed in this study and limits the generalizability of the findings to less educated subgroups within Arab immigrant populations.

The dissociation between acculturation’s positive correlation with trust (*r* = 0.228, *p* = 0.003) but negative association with satisfaction is particularly illuminating. This pattern suggests that these constructs operate through distinct psychological pathways - trust being primarily interpersonal and relationship-based, while satisfaction appears more sensitive to systemic and structural factors. This distinction has important implications for intervention strategies, suggesting that building trust with individual providers may not compensate for broader system- level deficiencies in the eyes of more acculturated patients.

The minimal explanatory power of sociodemographic variables (3.5% of variance) reinforces that satisfaction disparities stem primarily from mutable aspects of healthcare delivery rather than fixed patient characteristics. This finding does not support essentialist explanations of satisfaction gaps and instead points to the healthcare system’s responsibility in addressing these disparities. The sole significant demographic predictor - age - may reflect generational differences in healthcare expectations or life-stage variations in health needs.

Several theoretical frameworks help contextualize these findings. In line with Berry’s acculturation framework introduced earlier, different patterns of cultural adaptation may shape healthcare experiences in distinct ways [39]. The health literacy framework of Sørensen [18] helps explain how increasing health competencies might lead to more critical healthcare evaluations. Combining these perspectives, we propose a “critical health literacy” model for immigrant populations, where advancing health capabilities interact with cultural positioning to shape satisfaction judgments in complex ways.

### Practical implications

Practically, these findings suggest that improving patient satisfaction among Arab immigrants may require multilevel interventions operating at different points of care.

At the interpersonal level, trust-building could be supported through targeted training for doctors focusing on culturally sensitive communication, shared decision-making, and awareness of migration-related stressors. Such approaches could be integrated into existing continuing medical education formats without requiring major structural changes.

At the organizational level, doctors and primary care practices may benefit from strengthening language-concordant care and improving appointment accessibility, as difficulties with waiting times and scheduling were among the most frequently reported sources of dissatisfaction in this study.

At the systemic level, the findings suggest the relevance of addressing structural barriers that shape patient experiences beyond individual encounters. While not directly assessed in the present study, factors such as healthcare navigation support and institutional responsiveness to migrant needs may influence satisfaction, particularly among more acculturated and health-literate patients.

Finally, at the community level, health literacy initiatives tailored to migrant populations could move beyond information provision toward supporting patients in managing expectations and navigating complex healthcare systems. Such approaches may be most feasible when embedded within existing primary care or community-based structures.

The findings should therefore be interpreted with some caution, as non-response may be systematically related to patient experiences and perceptions of care.

## Conclusion

This study highlights the complex interplay of psychosocial and cultural factors shaping healthcare experiences for Arab immigrants in Germany. Trust in doctors emerged as the strongest positive predictor of patient satisfaction, while higher levels of health literacy and acculturation were paradoxically associated with lower satisfaction, suggesting that more informed and culturally adapted patients may critically evaluate systemic gaps in care. Importantly, these associations were observed across the subgroup distinctions examined in this study; however, they should be interpreted in light of the substantial heterogeneity within Arab immigrant populations.

The findings suggest the importance of multilevel interventions that prioritize trust-building through culturally sensitive provider training, address structural barriers such as accessibility and language services, and further refine health literacy initiatives to align with migrants’ evolving expectations and cultural identities. Future longitudinal and qualitative research is needed to further explore how acculturation processes and healthcare system interactions co-evolve over time to inform more equitable and responsive models of care for diverse migrant populations.

### Limitations

This study has several important limitations that should be considered when interpreting the findings.

First, the cross-sectional design prevents causal conclusions about relationships between acculturation, health literacy, trust in doctors, and patient satisfaction. Longitudinal analyses would be required to clarify temporal relationships and potential bidirectional effects.

Second, generalizability is limited by the online, snowball-based sampling strategy, which likely overrepresented younger, more digitally connected, and highly educated individuals; In particular, the sample included a high proportion of university-educated (65.5%) and married (60%) participants, which may not fully reflect the broader population of Arab immigrants in Germany. Marital status was not included in the regression models, and its potential influence on healthcare experiences could not be fully assessed.

Third, participants of Syrian origin constituted the majority of the sample, reflecting recent migration patterns in Germany but limiting the transferability of the findings to other Arab migrant groups. Although sensitivity analyses indicated that country of birth and humanitarian residence status were not independently associated with patient satisfaction, the available sample size did not allow for fully stratified analyses across distinct migration trajectories (e.g., forced vs. voluntary migration).

Fourth, several potentially relevant variables were not assessed. The acculturation measure did not distinguish between specific acculturation strategies (e.g., integration, assimilation, separation, marginalization), and additional factors such as perceived discrimination, prior trauma, healthcare expectations, and health insurance status were not included.

Additionally, health insurance status (private vs. statutory) was not assessed, limiting the ability to account for structural differences in access to care and healthcare utilization in the German healthcare system.

Fifth, all data were self-reported, which may introduce recall and social desirability bias. In addition, the EUROPEP instrument refers to healthcare experiences over the past 12 months, which may be subject to recall inaccuracies, particularly among individuals with infrequent healthcare utilization. Formal cross-cultural validation of some instruments, particularly in this specific population context, was beyond the scope of this study.

Sixth, migration-related timing was not captured in sufficient detail. Year of immigration was not collected, and duration of residence was used as an imperfect proxy. This may have introduced residual confounding, particularly given the close conceptual relationship between duration of residence, acculturation, and health literacy.

Finally, non-response bias and selection bias may have influenced the results. With a response rate of approximately 55%, it is possible that individuals who did not participate differ systematically from respondents in terms of healthcare experiences, satisfaction, or sociodemographic characteristics. In addition, the use of snowball sampling may have further contributed to selection bias, potentially overrepresenting individuals who are more highly educated, digitally connected, or engaged with healthcare-related topics. Consequently, more marginalized or less connected individuals may be underrepresented, which should be considered when interpreting the findings.

#### Future research

Future research should adopt longitudinal and prospective designs to examine temporal dynamics and potential causal pathways linking acculturation, health literacy, trust in physicians, and patient satisfaction. Tracking individuals over time would allow investigation of how satisfaction evolves across different stages of settlement and integration, and whether changes in language proficiency, social integration, or healthcare system familiarity influence these trajectories.

Mixed-methods approaches integrating quantitative surveys with in-depth qualitative interviews or focus groups would provide a richer understanding of migrants lived healthcare experiences. Qualitative data could help contextualize statistical associations, uncover culturally embedded expectations toward physicians, and identify structural or interpersonal barriers not captured through standardized instruments.

Future studies should also develop and validate more differentiated acculturation measures that distinguish between specific adaptation strategies (e.g., integration, assimilation, separation, marginalization) and assess bidimensional cultural orientation. Including constructs such as perceived discrimination, trauma exposure, healthcare expectations, and health insurance status would allow for a more comprehensive modeling of structural and psychosocial determinants of satisfaction.

Larger and more diverse samples are needed to enable fully stratified analyses across migration trajectories (e.g., forced vs. voluntary migration), legal statuses, duration of residence, and countries of origin. Collecting year of immigration and detailed migration histories would improve the examination of time-related effects and settlement processes. Comparative studies across different immigrant groups, as well as comparisons with non-migrant populations, could help disentangle culture-specific influences from more universal determinants of healthcare satisfaction within the German healthcare system.

## Supplementary Information

Below is the link to the electronic supplementary material.

Supplementary Material 1

## Data Availability

The survey data and original SPSS files are available from the corresponding author upon reasonable request.

## Declarations

### Letter to the editor

We would like to note that all Authors contributed equally to this work and should be considered co-first authors.

## Abbreviations

EUROPEP: European Project on Patient Evaluation of General Practice Care
UAE: United Arab Emirates
HLS-EU-Q16: European Health Literacy Survey Questionnaire (16-item short form)
BZgA: Federal Center for Health Education (Bundeszentrale für gesundheitliche Aufklärung)
SPSS: Statistical Package for the Social Sciences
SD: Standard Deviation
M: Mean
r: Pearson correlation coefficient
β: Standardized regression coefficient
SE: Standard Error
α: Cronbach’s alpha
B: Unstandardized regression coefficient
t: t-value
p: p-value
