# Supplementary material for: Health literacy, Trust in doctors, Acculturation - analysis of patient satisfaction of Arab immigrants in general medical care in Germany
Source: BMC Health Serv Res. 2026 May 5;26:675. doi: 10.1186/s12913-026-14675-z (PMC13159300; doi:10.1186/s12913-026-14675-z)

Supplementary Figure Legends
Supplementary Figure S1:
Normal P–P plot of regression standardized residuals for the dependent variable patient satisfaction.
Supplementary Figure S2:
Histogram of regression standardized residuals for the dependent variable patient satisfaction.

**FigureS1:**

**
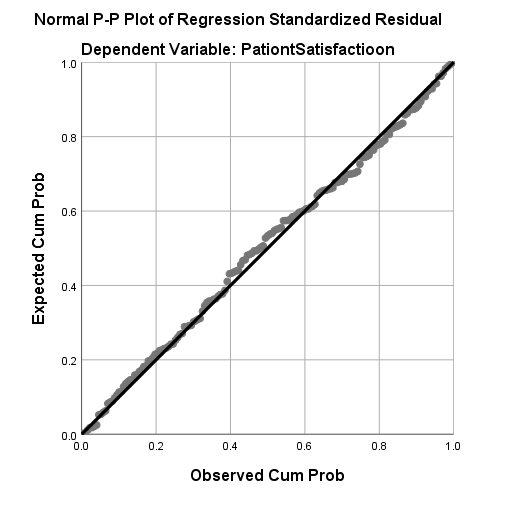
**

**Figure S2:**


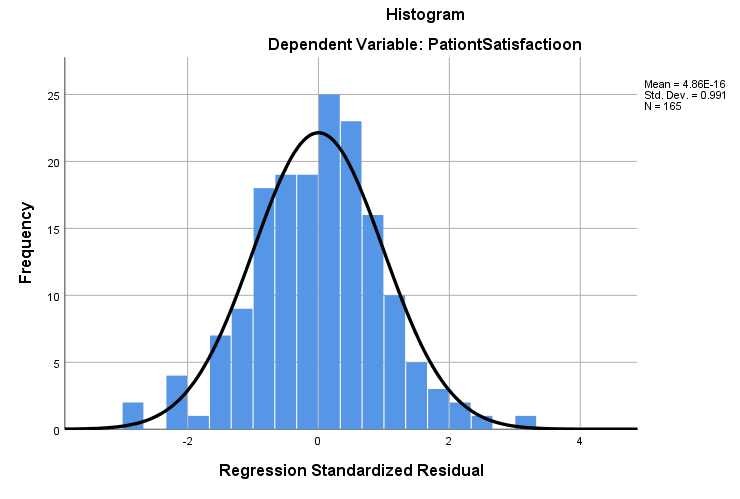

Supplement: Supplementary file 1 — Supplementary Material 1 [file 12913_2026_14675_MOESM1_ESM.docx]
